# Cervical cancer classification using a novel hybrid approach

**DOI:** 10.3389/fonc.2025.1703772

**Published:** 2025-12-04

**Authors:** Jheelam Mondal, Rajdeep Chatterjee, Mahendra Kumar Gourisaria, Manoj Sahni, Ernesto León-Castro

**Affiliations:** 1 School of Computer Engineering, Kalinga Institute of Industrial Technology (KIIT) Deemed to be University, Bhubaneswar, Odisha, India; 2 Department of Mathematics, Pandit Deendayal Energy University, Gandhinagar, Gujarat, India; 3 Faculty of Economics and Administrative Sciences, Universidad Católica de la Santísima Concepción, Concepción, Chile

**Keywords:** medical image analysis, cervical cancer, vision transformer, hybrid model, classification of images

## Abstract

**Objective:**

Cervical cancer is among the most frequently diagnosed malignancies in women. It is the fourth most prevalent malignancy in women worldwide. Pap smear tests, a popular and effective medical procedure, enable the early detection and screening of cervical cancer. Expert physicians perform the smear analysis, which is a laborious, time consuming and prone to mistakes. The main objective of our work is to distinguish or classify the healthy and malignant cervical cells using our proposed CASPNet model.

**Methods:**

This study proposes a novel technique by combining the concept of feature extraction by multi-head self-attention blocks, cross-stage partial network and feature fusion integration by spatial pyramid pooling fast layer components to identify healthy and cancerous cervical cells. Based on the comprehensive ablation study results, our proposed CASPNet architecture shows optimal performance having superior test accuracy with comparable computational efficiency.

**Results:**

The experimental study of our proposed model CASPNet (Contextual Attention and Spatial Pooling Network) has achieved an accuracy of 97.07% in the widely used benchmark SIPAKMED dataset.

**Conclusion:**

When compared to CNN models, self-attention blocks of vision transformer models are more accurate in classification tests and are generally better at capturing global contextual information inside an input image. The architecture’s CSP blocks are ideal for classification tasks with constrained resources where efficiency and speed are balanced; as a result, they are suitable for local feature extraction. Again, in cervical cells, objects are of varying sizes. Therefore, SPPF records contextual information at different receptive fields and performs multi-scale feature extraction. Hence, we can understand the images more precisely and reliably by incorporating all these benefits in our suggested CASPNet model.

## 1 Introduction

The fourth most common cancer among women is cervical cancer. Worldwide, cervical cancer has a serious impact on women’s lives and health. The World Health Organization reports that in 2022, almost 350 hundreds of women globally lost their lives to cervical cancer, while about 660 hundreds of women received new cervical cancer diagnoses. Cervical cancer continues to rank among the top causes of cancer-related mortality for women, especially in low- and middle-income nations (1). The Pap smear test is a commonly used and effective medical procedure that enables early identification and screening of cervical cancer. Accurate classification of cervical cancer cells is crucial for determining the stage and severity of the disease, which directly impacts therapeutic decisions and patient outcomes. However, traditional diagnostic methods can be time-consuming and prone to human error. To get past these challenges, current studies have concentrated more on combining machine learning and deep learning methods to improve classification’s precision, effectiveness and consistency. By automating the identification and categorization of abnormal cervical cells, these technologies offer promising solutions to improve screening programs and reduce mortality rates. As a result, various deep-learning-based methods have been created. Convolutional Neural Networks (CNNs), when thoroughly trained on extensive, well-annotated natural image datasets, have proved to be beneficial for disease diagnostic tasks, in spite of the differences between natural and medical images. Reusing the knowledge gained from one work for another is known as transfer learning, and it has been implemented by various researchers in classifying cervical cancer (2,3). Recently, vision transformer models (ViTs) have shown more remarkable performance than CNNs. Consequently, transformer-based models and their variants are effectively employed in numerous applications, including image classification (4). By switching from local convolutions to a global, data-driven attention mechanism, ViTs are excellent at feature extraction. Another essential and crucial component of You Look Only Once (YOLO) architecture is multi-scale feature learning. It enables models to recognize objects of various sizes with accuracy within an image. Hence a fundamental and distinguishing idea in YOLO models, is feature fusion integration. In our approach, we have combined the above concepts and created a new hybrid model by combining the ViT blocks of vision transformer model and CSP/SPPF blocks of YOLO to achieve the classification task. Then it is evaluated on the publicly available cervical cancer benchmark dataset, named SIPAKMED (5).

Further sections of the paper include Section 2 that discusses about the literature survey of cervical cancer cell classification, Section 3 comprises about the materials and methods used in the experimental study, Section 4 contains the discussion on results and analysis and also on the aspects of the proposed work to other related works and Section 5 covers the conclusion and future work.

## 2 Literature review

To improve the accuracy of a CNN model called AlexNet architecture, a padding strategy is implemented (1). The model’s accuracy is determined to have improved from 84.88% to 87.32%. The classification model performs well in identifying diseased cells with the classes of koilocytotic and dyskeratotic cells and normal cells with the classes superficial-intermediate and parabasal. Nevertheless, the model’s accuracy in identifying benign cell images with the class metaplastic is low. Again, using the ResNet-152 architecture, a deep learning classification method is developed that achieved a 94.89% classification accuracy on the SIPAKMED dataset. Prior studies have employed both machine learning and deep learning methodologies. Since machine learning approach captures the features manually, it takes up valuable time. Hence this study focuses on deep learning classification techniques only. In future, multiple datasets with comparable pap smear modality can be incorporated to make a more robust model. Avoiding the limiting environmental features is one advantage of doing this. Since time is crucial for diagnosing and determining the best course of treatment for cervical cancer, so training time can be also more optimized (2). (3) have used three pretrained models, InceptionV3, ResNet-50 and VGG19, to form the classification network. Out of these models, InceptionV3 has achieved highest accuracy value of 96.1%. In this paper, authors have also introduced visualization techniques for classification, which highlights the areas of an image that discriminate for a given class by computing an image-specific class saliency map. In future work, systematic integration of the image specific saliency maps into learning formulations will be done. (4) have applied an alternate approach of feature selection. Principal Component Analysis (PCA) and Grey Wolf Optimization (GWO) are used in this work. This work presents a two-level feature reduction technique that optimally selects feature sets by utilizing the benefits of both approaches. There is room for improvement through the use of hybrid metaheuristic feature selection algorithms and various classification models. This study paved a way for multi-domain adaptation and additional research in this area. Several classification problems, such as those in computer vision and biomedical applications, can be tested using the suggested process (10). An incremental deep tree (IDT) architecture is used by researchers to overcome CNN’s catastrophic forgetting in biological image classification. This framework enables CNNs to learn new classes while preserving accuracy on previously learned ones. Three well-known incremental methods are compared to the IDT framework to evaluate the efficacy of this strategy. The accuracy values attained by this method and using the SIPAKMED dataset is 93.00%. Since the suggested approach only affects the classifier’s output layer and ignores its internal architecture, experimenting with new deep models that optimize hyperparameters will enhance the outcomes (5).

A method is proposed that uses five polynomial (SVM) classifier models in an integrated cascade approach. The overall accuracy for all seven classes of the Herlev dataset is 97.3% and the test accuracy for all the classes is near to 92%. In this study, a method for automatically extracting features without the need for image processing techniques is proposed. In addition to reducing computing time, skipping this step can result in some image information loss, which would lower the accuracy of the proposed system. Additionally, it offers a high degree of confidentiality in order to quickly differentiate between various pap smear images (6). (7) has used a vision transformer module for global feature extraction and a convolutional neural network module for local feature extraction. The classification task is subsequently completed by fusing the local and global features using a multilayer perceptron module. Using the SIPAKMED dataset, this method reached a maximum accuracy of 91.72%. To increase the model’s effectiveness, further model combinations can be investigated in the future. Also, by altering the module architecture, feature extraction capability can be improved.

(8) have conducted a thorough investigation of two categories of the most sophisticated and promising deep learning techniques using over 20 vision transformer (ViT) models and 40 convolutional neural network (CNN) models. Additionally, the study uses data augmentation methods to change the data and ensemble learning methods to improve the model’s output correctness. The SIPAKMED dataset is used to test the methodology. For the classification task, EfficientNet-B6 achieves the best accuracy value of 89.95%, while ViT-B16 achieves the highest accuracy value of 91.93% among the ViT models. The ensemble approach that uses the EfficientNet-B6 and ViT-B16 models in conjunction with the max-voting process achieves the highest accuracy of 92.95%. (9) have used the Vision Transformer as the basis model for cervical cell classification and then incorporated an optimized pretrained MobileNet model to improve the output class prediction accuracy. The SIPAKMED dataset is used for this work and the accuracy value obtained is 97.65%. (10) have created the Cerviformer model by automatically classifying cervical cells using a cross-attention mechanism and a latent transformer model. This model can handle very large-scale inputs because it continuously combines the input data into a tiny latent transformer module using a cross-attention technique. With the SIPAKMED dataset, the maximum accuracy achieved with this method is 96.67%. (11) have conducted comparative analysis between vision transformer model variants and CNN model variants to classify the cervical cell images. It is observed that the vision transformer models outperform CNN models in terms of test accuracy. Using SIPAKMED dataset, highest accuracy of 93% is achieved with regularized ViT model variant named LeViT. To improve accuracy in the future, the ensemble technique that blends ViT models and CNN models can be investigated with reduced resource usage. Other cervical cancer datasets may also be used to investigate this model.

A more detailed analysis of the literature survey is again depicted in the following **Table 1**.

**Table 1:** Analysis of some research articles.

Author/Year	Method	Dataset used	Highest accuracy	Limitations
Haryanto et al./2020 (7)	AlexNet	SIPAKMED	87.32	The AlexNet architecture with a non-padding technique is used to create the CNN algorithm. Padding is applied to the source images by adding pixel 0 to improve the model accuracy. Though the overall accuracy of the model increased by 2.44% after applying padding scheme, for benign class Metaplastic, the accuracy value is only 54%.
Tripathi et al./2021 (8)	ResNet-152	SIPAKMED	94.89	Though deep transfer learning is applied in this study, the findings are limited to a specific dataset value only. In future, this model can be made more robust by combining several datasets with comparable pap smear modality. Since time is crucial for diagnosing cancer, this work can be designed more efficiently to consume less training time.
Dhawan et al./2021 (9)	InceptionV3	Intel MobileODT dataset	96.10	In future work, authors intend to more ethically integrate the image specific saliency maps into learning formulations.
Basak et al./2021 (10)	ResNet-50 +VGG16+Inceptionv3+ DenseNet121+PCA+ GWO	SIPAKMED	97.87	Ensemble technique based on different base learners is possible to explore in future.
Mousser et al./2022 (11)	IDT framework with CNN	SIPAKMED	93.00	There is a room for improvement by employing hybrid metaheuristic feature selection methods and other classification models. By creating an end-to-end multi-objective hybrid optimization technique that choose the ideal feature set, this feature selection can be further addressed.
Alquran et al./2022 (12)	SVM classifier	Herlev(7-class)	92.00	In addition to employing a high-performance CPU to handle a large amount of data, the system’s constraints can be addressed by adding additional datasets.
Liu et al./2022 (13)	CVM-Cervix (CNN+ViT+MLP)	SIPAKMED	91.72	Future research is to employ additional model combinations. The feature extraction capability can also be enhanced by changing the module structure. A variety of data pre-processing methods like color jittering and random crop can be used to improve the model’s capacity for generalization. Since capsule networks have recently been presented for image classification of learned features and may preserve spatial correlations of learned features, the performance of the suggested approach can be compared with that of a capsule network as part of future study.
Pascal et al./2023 (14)	ViT-B16+ max-voting EfficientNetB6+ max-voting	SIPAKMED	92.95 91.76	This approach can be utilized to create remedies for the diagnosis of different medical conditions.
Maurya et al./2023 (15)	ViT+CNN (simple averaging) LSTM+CNN	SIPAKMED	97.65 95.80	While the LSTM model takes significantly less CPU resources for training than ViT model, a higher classification accuracy has been achieved with ViT+CNN approach.
Deo et al./2024 (16)	Cerviformer (Cross-attention+ latent transformer)	SIPAKMED (3-class)	96.67	Future study is to focus on increasing the size of the dataset, so that overall accuracy can be kept consistent when working with images that have different degrees of dysplasia. Cofounding variables such as menstrual cycle, age can be added as new features to affect the output of the automated system. Also, future research with ThinPrep images can also be taken into account.
Mondal et al./2025 (17)	LeViT+ Regularization	SIPAKMED	93.00	Future study is to focus on ensemble strategy combining ViT and CNN architectures to build a robust classification system. This model can be explored on other cervical cancer datasets. The proposed approach can be used for classifying other medical diseases also.

## 3 Materials and methods

These resources and techniques used to categorize cervical cancer images are covered in this section. The experimental setup consists of the following steps: (i) dataset used for this study, (ii) dataset visualization (sample images of the dataset belonging to various classes), (iii) dataset augmentation and preprocessing, (iv) splitting of dataset (preparing the dataset in order to feed to the model), (v) proposed hybrid model (the model first processes the image patches through transformer blocks providing global feature learning through self-attention. Then reshapes the output to the CSP and SPPF components of YOLO for multi-scale processing and feature extraction and finally uses a classification head to predict the image class) and (vi) various software and hardware used for experimentation and (vii) different parameter values used in the hybrid model.

### 3.1 Dataset description

As per recent studies and literature survey, SIPAKMED dataset is a widely used standardized, publicly accessible with balanced classes and rich in morphology dataset used in the community. Since this dataset contains high intra-class variability and background artifacts, this dataset is comparatively challenging than others. The experimental study is implemented using the publicly available SIPAKMED dataset (6). 4049 isolated cell images in the.bmp format from 966 cluster cell images of pap smear slides make up this largest benchmark dataset. Expert cytopathologists have separated the cells into five different types based on their morphology and appearance, namely Parabasal, Superficial-Intermediate, Dyskeratotic, Koilocytotic and Metaplastic. Classes Parabasal and Superficial-Intermediate are considered as normal categories; Dyskeratotic, Koilocytotic and Metaplastic as abnormal categories. The number of pictures available in each class of the SIPAKMED dataset is shown in below **Table 2**.

**Table 2:** Class name distribution from the original SIPAKMED dataset.

Class name	Category	Number of cells
Superficial-Intermediate	Normal	813
Parabasal	Normal	787
Koilocytotic	Abnormal	825
Metaplastic	Abnormal	793
Dyskeratotic	Abnormal	813
Total		4049

### 3.2 Dataset visualization

**Figure 1:**
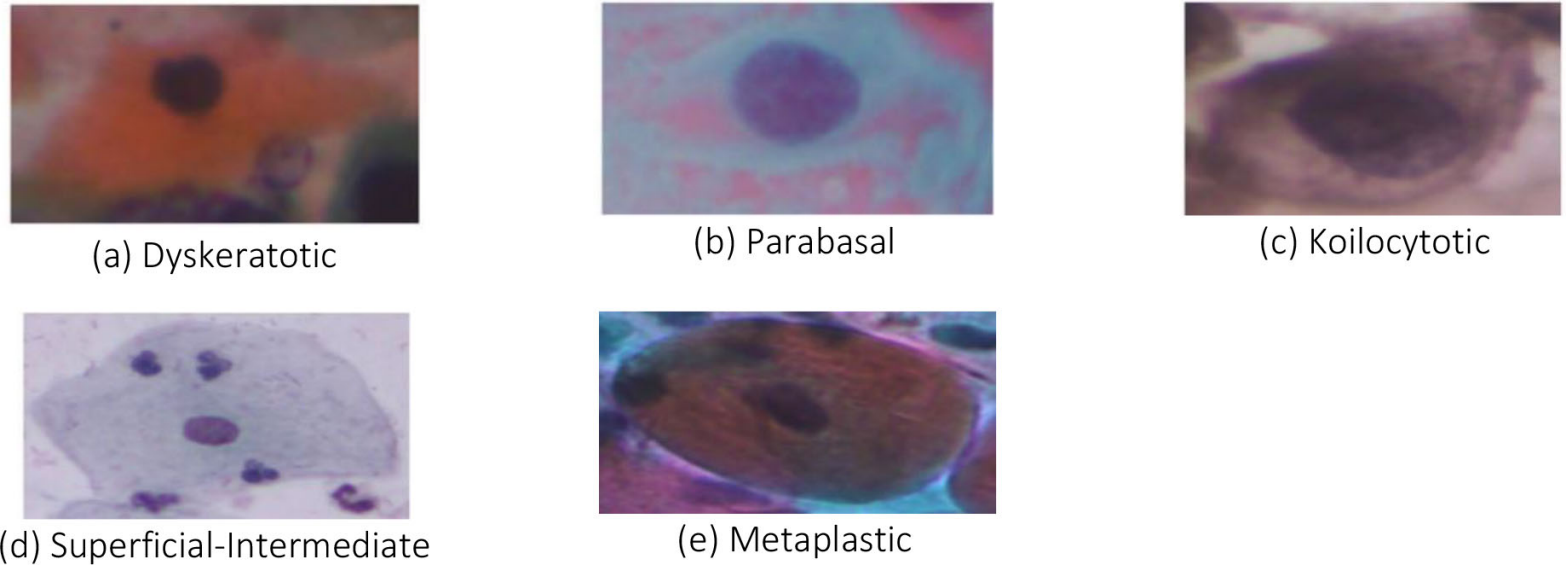
Sample images from each class in SIPAKMED dataset. **(a)** Dyskeratotic **(b)** Parabasal **(c)** Dyskeratotic **(d)** Superficial-Intermediate **(e)** Metaplastic.

The following **Figure 1** depicts the samples from each class in the widely used benchmark SIPAKMED dataset (12).

### 3.3 Data augmentation and image processing

The imbalanced class issue is resolved using the data augmentation technique. It aids in distributing data in a balanced manner. The number of images that fall into each class varies, as **Table 2** above illustrates. Data augmentation creates new images from old ones using a range of properties, such as cropping, flipping, rotating, and other methods. In this manner, the training dataset is made larger and of higher quality with the use of data augmentation. The various parameters considered for data augmentation are given in **Table 3**.

**Table 3:** Applied parameters for data augmentation.

Parameters	Values
RandomResizedCrop	224
RandomHorizontalFlip	0.5
RandomVerticalFlip	0.5
RandomRotation	20
ColorJitter	saturation=0.1, hue=0.1, brightness=0.1, contrast=0.1

### 3.4 Splitting data

To make sure that each set is representative of the entire dataset, it is essential to randomize the data before splitting it. This reduces the possibility of bias in the model’s evaluation and training. Stratification guarantees that each set retains the same percentage of classes as the original dataset in classification task. This is especially crucial when working with imbalanced datasets. Therefore, for training, validation, and testing purpose, the dataset is split into 80:10:10 ratio using the holdout method as part of a uniform data partitioning strategy to guarantee experimental consistency. Additionally, in order to preserve the reproducibility of the outcomes in a single run, a fixed random seed value of 1337 is considered.

### 3.5 Proposed hybrid classification model

**Figure 2:**
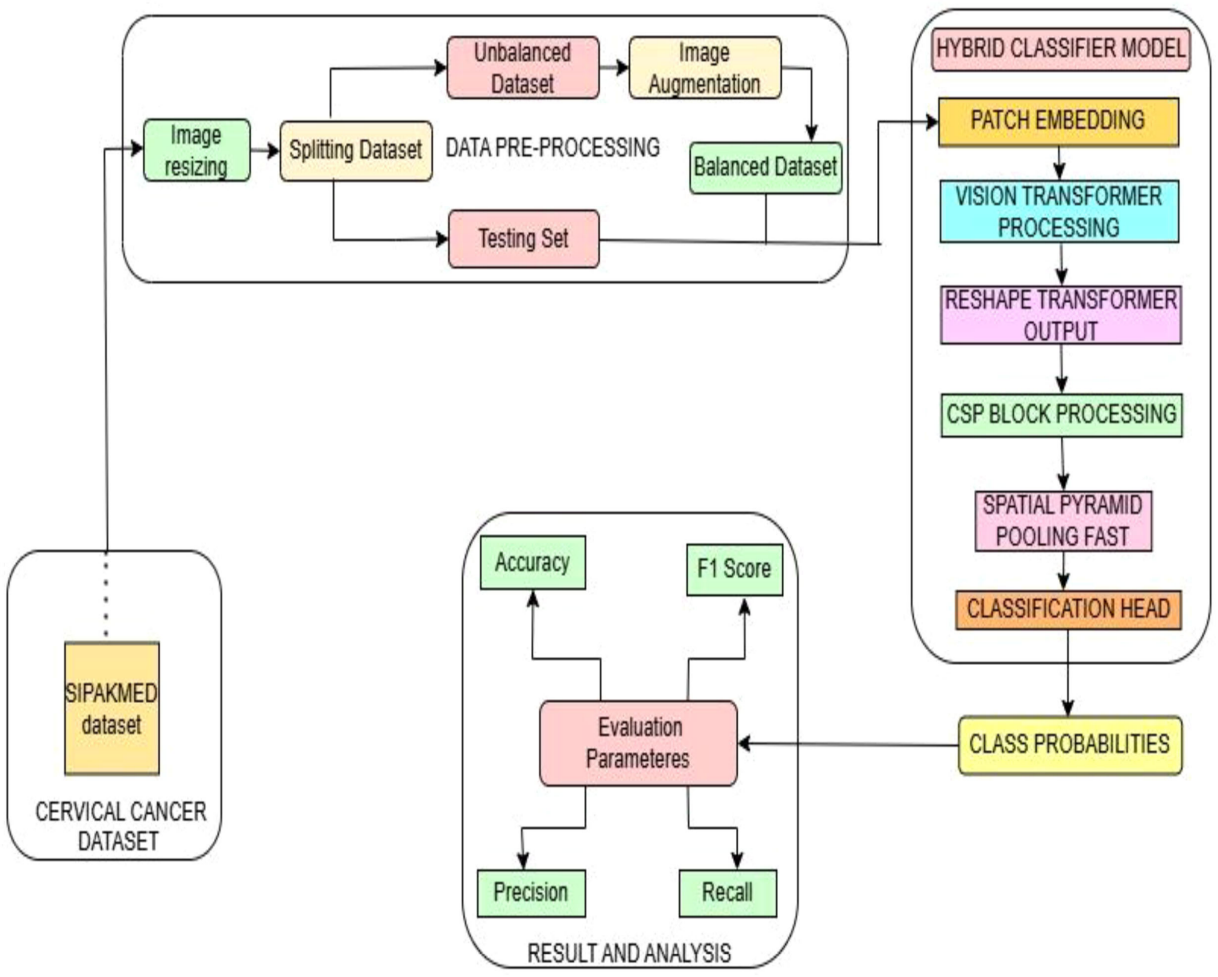
Proposed methodology workflow for classifying cervical cells.

The following **Figure 2** shows the proposed overall workflow of our experimental study for classifying cervical cancer. The proposed classification model is a hybrid model architecture that combines Vision Transformer (ViT) with YOLO components. In **Algorithm 1**, the key components of this hybrid classifier include the following:

1) Patch Embedding Layer: The input image is divided into patches and projected into an embedding space in this layer.
2) Vision Transformer blocks: It provides global feature learning through self-attention mechanism.
3) Cross Stage Partial (CSP) blocks: This is a YOLO component that is used for efficient feature extraction. It is achieved by splitting the feature map of a base layer into two parts, which are then combined using a cross-stage hierarchy.
4) Spatial Pyramid Pooling Fast (SPPF): is used from YOLOv5 for multi-scale feature maps representation. To enhance the model’s capacity to capture features at various levels of abstraction, this component is used.
5) Classification head: After feature extraction layers of the model, normalization of the layer is done to stabilize the training, and then a linear transformation is applied to the input to map the high-dimensional space to the number of classes to predict each class logits.

Hence, this new hybrid model first processes the image patches through transformer blocks. It then reshapes the output to operate with the CNN-based YOLO components. Lastly, a classification head is used to predict the class probabilities.

Algorithm 1 **Hybrid CASPNet model for cervical cancer image classification.**

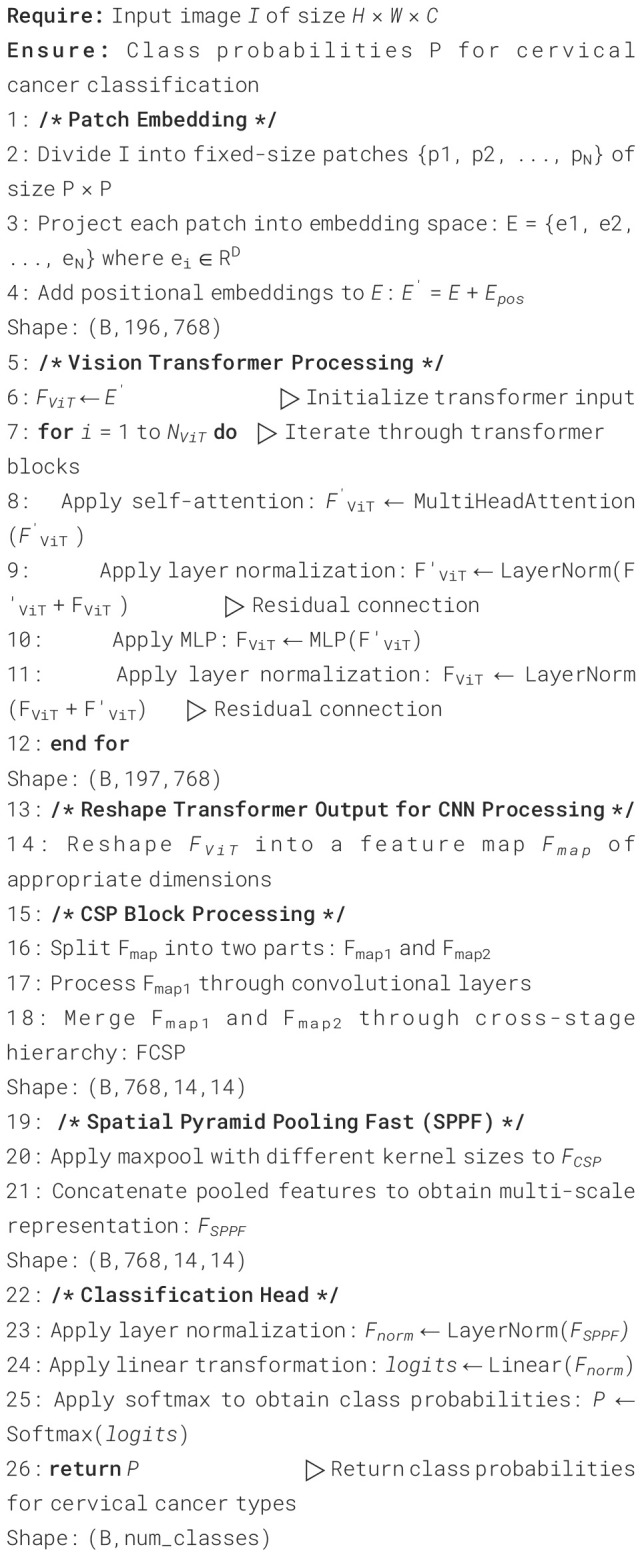

The specification tables for our hybrid classifier are given below in **Tables 4**–**7**. **Table 4** contains Structure of Input Processing, **Table 5** describes structure of transformer block. **Table 6** and Table details about structure of CSP Block and SPPF Block.

**Table 4:** Structure of input processing.

Stage	Operation	Parameters	Output shape
Input	RGB Image	in channels = 3	(B,768,14,14)
Patch Embedding	Conv2d + LayerNorm	patch size=16, embed dim=768	(B,196,768)
Position Embedding	Add CLS Token + Pos Embedding + Dropout	num_patches=196, p=0.1	(B,197,768)

**Table 5:** Structure of transformer blocks.

Component	Details	Parameters
Block structure	Each of 12 blocks	depth=12, num_heads=12, mlp_ratio=4.0
		qkv_bias=True, drop=0.1, attn_drop=0.1
Attention Layer	X=x+Attention(LayerNorm(x))	heads=12, head_dim=64, scale=0.125
	Multi-Head Attention	Q,K,V= Linear(768->786 X 3) with bias
		Attn = softmax
MLP Layer	X=x+MLP(LayerNorm(x))	768 –> 3072 -> 768, GELU, drop=0.1

**Table 6:** Structure of CSP block.

Layer	Operation	Parameters	Output shape
Split	Split channels	in_channels=768, out_channels=768	(B,768,14,14)
Branch1	Conv2d	768 -> 384, k=1, s=1	
Branch2	Conv2d + Transformer	768 -> 384 + Transformer	

**Table 7:** Structure of SPPF block.

Layer	Operation	Parameters	Output Shape
Input		in_channels=768, out_channels=768	(B,768,14,14)
Conv1	Conv2d	768 -> 384, k=1, s=1	
MaxPool2d	3 cascades	k=5, s=1, p=2	
Concat	Concatenate pooling outputs		
Conv2	Conv2d	1536 -> 768, k=1	
Output	Spatial Pyramid Pooling Fast		(B,768,14,14)

### 3.6 Detailed architecture of the hybrid classification model

**Figure 3:**
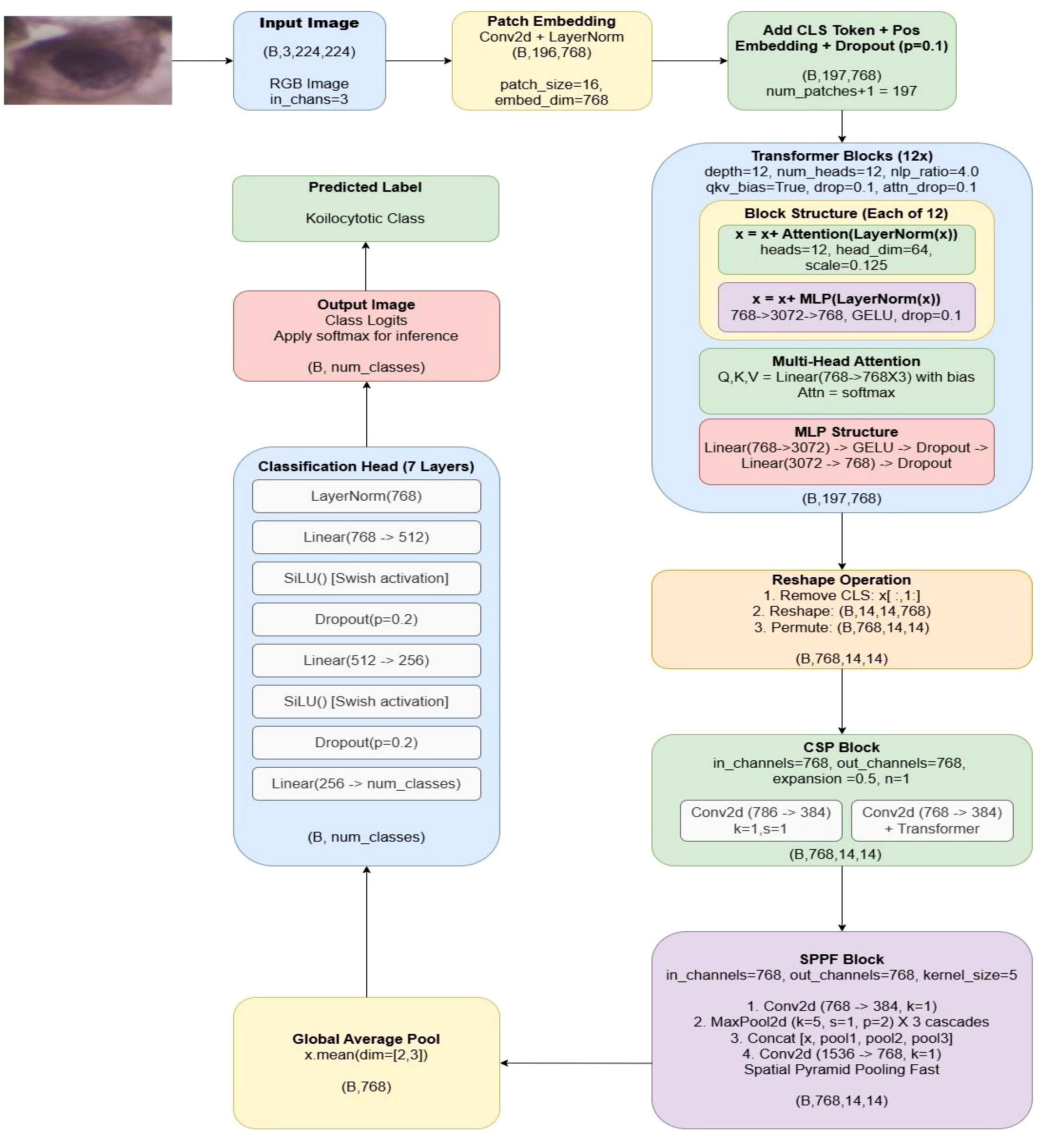
Detailed diagram of the proposed CASPNet model for cervical cancer diagnosis comprising basic three components: patch embedding and transformer blocks, CSP block, and SPPF block.

Following, **Figure 3** depicts the detailed architectural diagram of the proposed CASPNet hybrid model for cervical cancer image classification. Our CASPNet model is a hybrid model where vision transformer blocks are integrated with CNN components namely Cross-Stage Partial (CSP) and Spatial Pyramid Pooling Fast (SPPF) blocks. Bridging the dimensional gap between the spatial feature maps required by convolutional layers and the sequence-based representations of transformers is a crucial problem in such hybrid system. The model applies to a patch size of 16 pixels. The patch embedding layer transforms the input image of 224x224 with 3 RGB channels into a flattened output as (B,196,768). Following path embedding, inside vision transformer processing, to encode spatial information, learnable positional embeddings are inserted, and a learnable class token is appended to the sequence. This sequence passes through 12 transformer blocks to keep the output as (B,197,768), where B is the batch size, N is the number of tokens (197) and D is the embedding dimension (768). After this, the model does not introduce any learned projection layers. Instead, it uses inverse patch embedding (geometric reshape) operation to convert the transformer’s sequence representation to the spatial feature maps needed by CSP and SPPF blocks. Since no trainable parameters are added in this approach, it also reduces model complexity and avoids overfitting. The reshaped tensor (B,768,14,14) is an appropriate 4D tensor format for convolutional operations inside CSP and SPPF blocks. Local-global feature fusion is enabled in CSP block. The SPPF block uses cascaded max pooling to aggregate multi-scale information while preserving spatial dimensions. As a spatial aggregation technique, the global average pooling operation preserves the channel-wise feature representations. The output now becomes (B,768) by calculating the arithmetic mean for each channel separately across the two spatial dimensions, width and height. Finally, the classification head uses a hierarchical, fully-connected network with non-linear activations and regularization to change the 768-dimensional feature vector into class probabilities.

The below **Table 8** depicts the hyperparameter table containing the parameters used by ViT block. Whereas **Table 9** below shows the dimensionality transformation strategy taken by CSP block from the ViT output. As our model does not introduce any learnt projection layers since convolutional projection layers such as nn.Conv2d, nn.ConvTranspose2d or nn.Linear are not included between the ViT and CSP blocks. Rather, it employs geometric reshaping, or inverse patch embedding. This transformation is parameter-free and relies on the spatial structure of the patch tokens being preserved.

**Table 8:** ViT block.

Parameter	Value	Comments
Depth	12	Transformer block number
Head Number	12	Multi-head attention heads
MLP Ratio	4.0	Hidden dimension expansion in MLP
QKV Bias	True	Bias in query/key/value projections
Dropout Rate	0.1	General dropout rate
Attention Dropout	0.1	Dropout in attention mechanism
Position Embedding	Learnable	Shape: (1,197.768)
Class Token	Learnable	Shape: (1,1,768)

**Table 9:** ViT output to CSP input (dimensionality transformation strategy).

Stage	Dimension	Shape	Comments
ViT output (with CLS)	Sequence	(B,197,768)	1 CLS token + 196 patches
Remove CLS Token	Sequence	(B,196,768)	X[:1]: discards first token
Reshape to Spatial	4D Tensor	(B,14,14,768)	reshape (B,14,14,768)
Permute to Conv format	4D Tensor	(B,768,14,14)	permute (0,3,1,2)

### 3.7 Software and hardware used

The experiments have been implemented with PyTorch (2.5.1) and TorchVision (0.20.1) compiled with CUDA version 12.4 on a Google Colab Notebook. The hardware included an i5 11th generation processor and 16 GB RAM. We have used NVIDIA T4 GPU which is a powerful and energy-efficient GPU that speeds up processes related to computer vision.

### 3.8 Model parameters used

The proposed model is run from scratch for 450 epochs using a learning rate value of 0.001 and a weight decay value of 0.05. CrossEntropyLoss is used as the loss function. OneCycleLR scheduler is used in the experimental setup to increase the neural network training speed and efficiency. For better performance, the optimal batch size considered is 128. All the above parameters used along with other parameters considered in our proposed CASPNet model are listed in **Table 10** and are discussed below.

**Table 10:** Hyperparameters used for experiments.

Hyperparameters	Values
Rate of Learning	0.001
Decaying Weight	0.05
Loss Function	CrossEntropy
Batch Size	128
Epochs	450
Size of Image	224 X 224 X 3
Optimizer	AdamW

Learning Rate is a scalar variable and is the step taken in the direction of the negative gradient during backpropagation. Backpropagation is the technique of updating the weights of a neural network by propagating the error between the expected and actual outputs backward through the network. A one cycle learning rate policy is used by the scheduler in the experimental study. Using a cosine annealing technique, it begins with a low learning rate (0.0001), raises it to a maximum of 0.001 during the first 10% of training and then progressively lowers it a very low value (0.00001) at the end of training.

CrossEntropyLoss function is common in neural networks for multi-class classification. It is crucial in evaluating the probability of the predicted class labels matching the actual class labels. The multi-class classification statement is shown in the following Equation 1.

(1) $$Loss=-\sum_{c=1}^{C}y_{i,c}*\text{log}\left(p_{i,c}\right)$$

Here, C represents the total number of classes, $y_{i,c}$ denotes the true label for class c and $p_{i,c}$ denotes the predicted probability of class c.

Neural networks employ weight decay as a regularization strategy to prevent overfitting. To keep the model’s weight as low as possible, a term is added to the loss function. Usually, the L2 norm of the weights serves as this penalty term. The weight decay equation is expressed in the following Equation 2.

(2) $$Loss_{total}=Loss_{original}+\lambda *{\left|\left|W\right|\right|}^{2}$$

Here, $Loss_{total}$ is the sum of the original loss and the weight decay penalty, $Loss_{original}$ denotes the original loss function, λ represents the weight decay coefficient, W represents the vector of model weights and ${\left|\left|W\right|\right|}^{2}$ is the L2 norm of the weights which gives the total of the weights squared.

Because it directly affects the accuracy and computing efficiency of the training process, batch size is one of the most crucial hyperparameters in deep learning training. It indicates the quantity of data used in a single forward and backward trip through the network. In our study, a batch size of 128 is considered to train the neural model.

The image size refers to the dimensions of the input images that are given into the deep learning model during training. The image’s height and width are represented in pixels by 224. The image’s color channel count is represented by 3. Typically, this represents the RGB color images.

Another important aspect is the epoch, where an epoch corresponds to a whole run through the entire dataset. The model views each training example once throughout each epoch and modifies its parameters based on the loss function. In our experimental study, we have observed that the test accuracy has increased after every iteration, resulting in a graph that showcases that the scratch model is able to learn the dataset gradually after each epoch. A total of 450 epochs have been considered for training the model with the dataset and reported single run results with seed value as 1337. To check for robustness of the model, we have done strict cross validation.

Lastly, the optimizers play an important role while training the model. In deep learning, optimizers are algorithms that modify the weights and biases of the model in order to minimize the loss function. They control the network’s data-driven learning process. The AdamW optimizer is a modification of Adam, where it implements the weight decay correctly and has better generalization in many cases. It is particularly effective for transformer models. Given parameters are *θ*, Loss function L(*θ*), Learning rate *η*, Weight decay coefficient *λ*, Exponential decay rates *β*_1_, *β*_2_ ∈ (0, 1), small constant *ϵ* to prevent division by zero. And the AdamW optimizer’s general parameter update given below in mathematical Equation 3.

(3) $$\theta \left(t+1\right)=\theta \left(t\right)-\eta .\hat{m}\left(t\right)/\sqrt{\hat{v}\left(t\right)}+\epsilon \;-\;\eta .\lambda .\theta \left(t\right)$$

Here, model parameters updated at a given time step t is denoted by θ(t). Rate of learning is denoted by η. The bias-corrected first-moment estimate is $\hat{m}\left(t\right)$. Bias-corrected second-moment estimate is $\hat{v}\left(\text{t}\right)$. ϵ is a small constant added to denominator to keep numerical stability and the weight decay coefficient is denoted by λ. We have done rigorous experiments by using different optimizers and have found that AdamW optimizer performance is much better for SIPAKMED dataset. The reason is that the SIPAKMED dataset is a complex dataset with high inter-class similarity and AdamW optimizer excels with such complex, similar classes.

## 4 Results and analysis

This section discusses the ablation analysis and conclusions pertaining to the experimental findings. The most widely used and recognized evaluation metrics in classification task studies are accuracy, recall, precision and F1-score (18, 19).

### 4.1 Ablation study

The suggested CASPNet (ViT+CSP+SPPF) architecture exhibits optimal performance across several assessment parameters based on the thorough ablation study findings, confirming the integration of its component parts. Following **Table 11** displays the ablation study experimental results. With a test accuracy of 97.07%, the complete model outperforms all ablated variants by 30.62% over the standalone ViT architecture (66.45%), 75.85%% over CSP+SPPF alone (21.22%) and 14.84% over ViT+CSP without SPPF (82.23%). These findings show that each architectural element provides crucial complementary capabilities; SPPF module aggregates multi-scale spatial features through pyramidal pooling, the CSP block improves gradient flow and feature reuse through cross-stage partial connections and the vision transformer offers global context modeling through self-attention mechanisms. It is found that, the ViT-only configuration has a poor test accuracy and severe underfitting despite having the fewest parameters (46,277) and computational cost (23.081M FLOPs). This suggests that pure transformer architectures do not have enough inductive biases for successful visual feature extraction in this domain. The CSP+SPPF module on the other hand, shows catastrophic overfitting despite moderate computational requirements (4.765G FLOPs), achieving only 21% test accuracy while requiring total training time of 2273.911 secs. This suggests that long-range dependencies required for robust classification cannot be captured by convolutional components alone. Although ViT+CSP module requires the longest training time (9831.581 secs) and largest computational cost (17.442G FLOPs) among partial architectures, it achieves fair performance (82.23% test accuracy) but still falls short of the CASPNet model.

**Table 11:** Ablation study results.

Method	Total model parameters (millions)	Computational cost (FLOPs)	Test accuracy (%)	Total training time (seconds)
ViT+CSP+SPPF	90.755	17.731G	97.07	9972.122
ViT	0.046	23.081M	66.45	1511.14
CSP+SPFF	37.212	4.765G	21.22	2273.911
ViT + CSP (No SPPF)	89.281	17.442G	82.23	9831.581

The complete ViT+CSP+SPPF module achieves the best training time (9972.122 secs) and superior test accuracy with comparable computational efficiency (17.731G FLOPs) and having 90.76M total trainable parameters, proving that the SPPF module improves convergence properties in addition to representational capacity. To ensure fair comparison and attribute performance differences only to architectural modules, all setups has used the same hyperparameters (learning rate value as 0.001, AdamW optimizer and 450 epochs). Hence the complete ViT+CSP+SPPF architecture is the best configuration for this cervical cell classification task because it strikes the ideal balance between global context modeling, hierarchical feature extraction and multi-scale spatial aggregation, as demonstrated by the ablation study results.

### 4.2 Our hybrid model performance on SIPAKMED dataset

In our experimental study, we have considered the performance metrics as mentioned below in **Table 12**. **Table 12** contains the equations corresponding to the evaluation parameters considered. The first important aspect of measuring the performance of a classification model is its accuracy. It is a simple method to evaluate the model’s overall performance. The formula depicts the proportion of the number of correct predictions to the total number of predictions. Hence, a greater number of values of true positives (TP) and true negatives (TN) will lead to a greater accuracy of the model.

**Table 12:** Performance metrics used for experiments.

Performance metrics	Formula
Accuracy	$\frac{TP+TN}{TP+TN+FP+FN}$
Precision	$\frac{TP}{TP+FP}$
Recall	$\frac{TP}{TP+FN}$
F1-score	$\frac{2*P*R}{P+R}$

The next crucial performance metric of a classification model is precision. It focuses on the accuracy of positive predictions. Therefore, it is represented as the percentage of accurately predicted positives out of all predicted positives. It is especially crucial when the expense of false positives is significant. In medical diagnosis, since a false positive case might lead to improper treatment decisions, hence in such scenario precision plays an important role in the model’s performance. Also, in imbalanced dataset cases where one class greatly outnumbers the other, precision is extremely important. Alone, accuracy cannot be treated as a performance metric for the overall performance of the model.

Recall, or sensitivity, is also a crucial performance parameter, especially in classification tasks. The ability of the model to locate all real positive cases is the main focus of recall. In medical diagnosis problems, missing a positive instance (false negative) has a significant cost, hence recall is essential. Meaning, if a person who actually has cervical cancer is not detected as a cancer patient, then it will have severe consequences. Because positive cases are uncommon in imbalanced datasets, recall is very crucial in judging the models’ performance. In these situations, accuracy alone can be misleading; hence the precision parameter is taken into account.

Recall and precision are often traded off in real-world situations. Low recall can result from high precision and vice versa. Finding a balance between these two is aided by the F1-score. In the case of imbalanced datasets, even though the model is highly accurate, it would be pointless if it consistently predicted the majority class. In these situations, the F1-score metric provides a more precise representation of the model’s performance. It is denoted as the harmonic mean of precision and recall.

**Figure 4:**
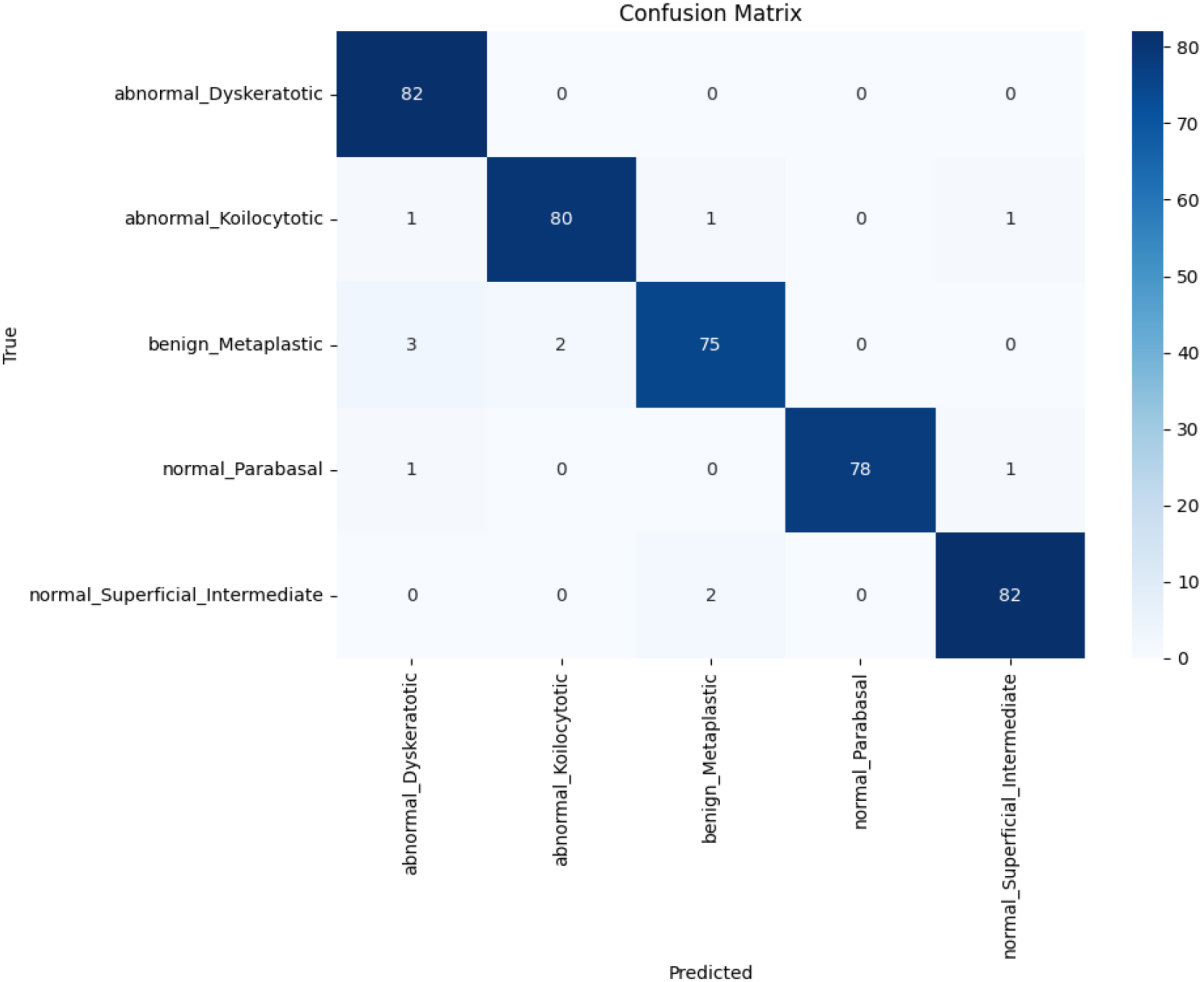
Confusion Matrix (CM) on SIPAKMED dataset.

The confusion matrix table present above provides an overview of a classification algorithm’s performance. The number of true positives, true negatives, false positives, and false negatives are shown in this table and it shows how well a classification model performs by comparing its predicted labels to the actual labels. The below **Figure 4** displays the confusion matrix generated using our proposed model on the SIPAKMED dataset.

It shows that there are 397 true cases out of 409 test cases which use the SIPAKMED dataset. Our proposed CASPNet model demonstrates its robustness by overcoming challenges such as inadequate data, image fluctuations, and image quality problems. This very feature of our model demonstrates its usefulness in real-time cervical cancer diagnosis. As observed in **Figure 4**, our scratch CASPNet classifier model achieves 3 FN values for the Koilocytotic class, 5 FN values for the Metaplastic class, 2 FN values for the Parabasal class, and 2 FN values for the Superficial Intermediate class while correctly diagnosing all the images of the Dyskeratotic class. The following **Table 8** shows the performance metrics of the proposed model across the SIPAKMED dataset.

**Figure 5:**
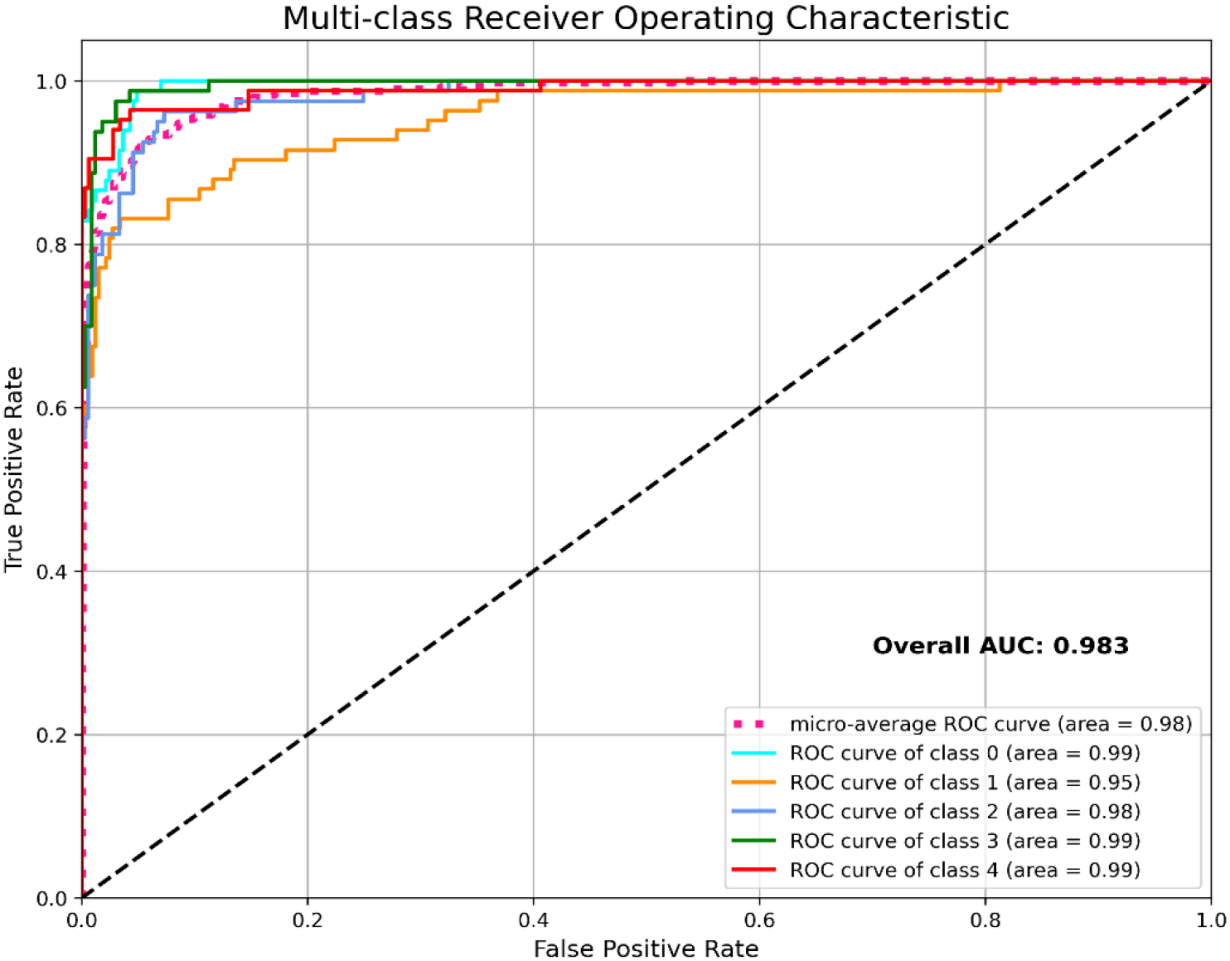
ROC-AUC curve on proposed CASPNet model using SIPAKMED dataset.

Again, **Figure 5** displays the ROC-AUC graph that contrasts the True Positive Rate (TPR) or sensitivity and False Positive Rate (FPR) or (1-Specificity) at various categorization criteria. It is a technique to evaluate a model’s capacity for discriminating input based on class.

The following Equations 4, 5 denote the formulas corresponding to TPR and FPR respectively.

(4) TPR=TP/(TP+FN)

Here, true positives are denoted by TP and false negatives by FN.

(5) FPR=FP/(FP+TN)

Here, FP denotes false positives and TN denotes true negatives.

The following **Table 13** displays the accuracy per class of the proposed scratch model achieved when applied on the SIPAKMED dataset. It is observed that, Metaplastic category has the lowest accuracy rate due to its morphological ambiguity and inter-class similarity between Parabasal and Koilocytotic classes. So, even when using deep learning architectures, metaplastic cells are intrinsically difficult for feature discrimination due to the fluctuating nuclear-to-cytoplasmic ratio and irregular chromatin patterns.

**Table 13:** Per-class accuracy using the proposed scratch model on SIPAKMED dataset.

Class name	Accuracy/Class (%)
Dyskeratotic	100.00
Koilocytotic	96.39
Metaplastic	93.75
Parabasal	97.50
Superficial Intermediate	97.62

### 4.3 Explainability and interpretability

In computer vision, Grad-CAM (Gradient Weighted Class Activation Mapping) is a potent method for comprehending and visualizing the reasons behind a convolutional neural network (CNN) prediction. Grad-CAM creates a heatmap, which is a data visualization in which values are represented by colors. Red colors show the areas of high interest or high activation, whereas blue colors represent areas of low importance or low activation. This is accomplished by calculating the gradients of the target class’s score with respect to the feature mappings of the final convolutional layer. These gradients show how significant each feature map is to the prediction. It creates a heatmap, which is a data visualization in which values are represented by colors. Hence, Grad-CAM assists in determining whether the model is concentrating on background noise or pertinent features. In our approach, the SPPF layer satisfies the fundamental requirements for Grad-CAM application by maintaining spatial feature representations and differentiable routes during backpropagation, even though it uses max-pooling procedures. Below, figures indicate questionable spots or abnormalities on pap smear slide images, allowing medical professionals to identify potential cervical malignancies. In this sense, explainability is essential so that medical professionals may review the logic of the model and ensure that it aligns with medical knowledge.

**Figure 6:**
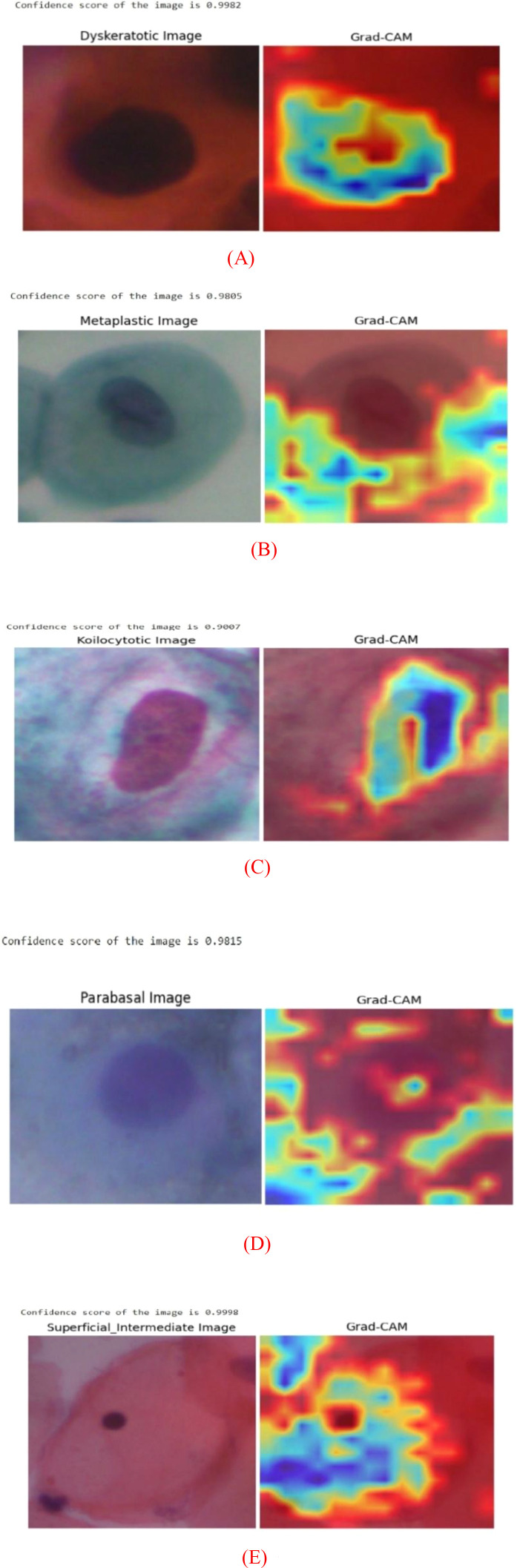
Grad-CAM results on various class images of SIPAKMED dataset.

Above, **Figure 6** shows the Grad-CAM results of sample input images belonging to a different class image of SIPAKMED dataset. **Figure 6A** shows a single dyskeratotic type of squamous cell from the SIPAKMED dataset, with a high nucleus-cytoplasm (N:C) ratio, irregular nuclear membrane, and hyperchromatic nucleus. This shows that the heatmaps give special attention to the aberrant features of the cell’s nucleus and the surrounding dense, aberrantly keratinized cytoplasm. Hence it indicates that the CASPNet model focuses on abnormal nuclear features. **Figures 6B–E** presents the other class images present of the dataset and their corresponding Grad-CAM results with high confidence scores. It is obvious from the outputs that our model is successfully applying the fundamental, valuable diagnostic cues that human pathologists utilize to detect cancerous cells. This robust spatial alignment demonstrates that the CASPNet model does not rely on artifacts or erroneous background characteristics.

### 4.3 State-of-the-art study

On the SIPAKMED dataset, the suggested CASPNet model has an accuracy of 97.07% and a weighted average F1 score of 97% at epoch number 402.

Furthermore, several of the previously proposed methods for categorizing pap smear cervical cell images have been compared with the approaches detailed in this paper. The experimental results of the current study are contrasted with those of previous approaches in **Table 14**. The effectiveness of the suggested approach is therefore demonstrated by its strong performance on a variety of performance metrics. The suggested approach performs better and is, in some cases, competent with current state-of-the-art techniques, as indicated in **Table 14**.

**Table 14:** Comparison with related work using cutting edge methods for SIPAKMED dataset.

Author name/year	Model name	Accuracy (weighted average) (%)	Precision (weighted average) (%)	Recall (%)	F1-score (weighted average) (%)
Haryanto et al./2020 (1)	AlexNet	87.32	–	–	–
Tripathi et al./2021 (2)	ResNet-152	94.89	95.80	95.00	95.40
Basak et al./2021 (4)	ResNet-50 +VGG16+Inceptionv3+ DenseNet121+PCA+GWO +SVM classifier	97.87	98.56	99.12	98.89
Mousser et al./2022 (5)	IDT framework with CNN	93.00	–	–	–
Liu et al./2022 (7)	CNN+ViT+MLP	91.72	91.80	91.60	91.70
Pascal et al./2023 (8)	ViT-B16+max-voting	92.95	–	–	93.30
Maurya et al./2023 (9)	ViT+CNN (simple averaging)	97.65	–	–	–
Deo et al./2024 (10)	Cross Attention + Latent Transformer	96.67	–	–	–
Mondal et al./2025 (11)	LeViT+ Regularization	93.00	–	–	–
Our proposed CASPNet model (2025)	**Scratch Model**	**97.07**	**97.00**	**97.00**	**97.00**

### 4.4 Discussion

Our suggested architecture, which combines ViT, CSP and SPPF components, shows exceptional efficacy in capturing morphological characteristics of cervical cells for classification task, achieving 97.07% accuracy. Given that the model is trained from scratch, this performance is quite noteworthy and shows how well our architectural design decisions work. Our model has attained a commendable accuracy of 97.07%, representing only a marginal 0.58% difference from the pretrained baseline of Maurya et al. (15) work and a marginal difference of 0.80% from pretrained baseline of Basak et al. (10) work. Maurya et al. has used pretrained models ViT/L32 and MobileNetV1 whereas Basak et al. work has applied pretrained models VGG16, ResNet50, Inceptionv3, DenseNet121 ensemble model. Such pretrained models are already trained on huge ImageNet database. Our well-constructed scratch model, which includes elements like ViT, CSP and SPPF, shows the model’s strong capacity to recognize and categorize cervical cell morphological characteristics. This itself is a novel algorithm and it includes a lot of human effort and is also a time-consuming process in terms of building the model architecture meticulously. The suggested CASPNet model having 17.731 GFLOPs performs similarly to the approach described by Maurya et al. (15), which attained 15.581 GFLOPs, in terms of computational complexity as measured by GFLOPs. However, compared to the ensemble-based method suggested by Basak et al. (10), which shows a noticeably larger result of 28.261 GFLOPs, it necessitates significantly fewer calculations. **Table 15** summarizes the comparative experimental results across several important factors.

**Table 15:** Comparison with best SOTA works.

Author name/year	Method	GFLOPs	Transfer learning	Observations
Basak et al./2021 (4)	VGG16+ResNet50+Inceptionv3+ DenseNet121 (excluding PCA + GWO)	28.261G	Yes	This paper uses pretrained models whose total features is reduced further by PCA followed by an optimization technique.
Maurya et al./2023 (9)	ViT/L32 + MobileNetV1	15.581G	Yes	This study makes use of pretrained models ensemble concept.
Our Work (2025)	ViT+CSP+SPPF	17.731G	Scratch Model	It is a new novel algorithm. It is a very thoughtful and clever written algorithm rather than a haphazard integration.

## 5 Conclusion and future work

Our experimental study represents a significant turning point in the field of cervical cancer image categorization using state-of-the-art deep learning techniques and vision transformer models. Combining self-attention, cross-stage partial network (CSP) blocks and spatial pyramid pooling fast (SPPF) layer components, our model architecture is thereby adapted for image classification. This specifically shows the innovative changes made in this study, spurring an advancement in this area. Self-attention blocks of vision transformer models excel in capturing global contextual information in general within an input image, and they have improved accuracy in classification tasks compared to CNN models. The CSP blocks in the architecture are perfect for classification task with limited resources where speed and effectiveness are balanced; hence, it is appropriate for local feature extraction, resulting in real-time applications. Again, in cervical cells, objects are of varying sizes. So, multi-scale feature extraction is done by SPPF and it records contextual information at various receptive fields. Hence, combining all these advantages in our proposed CASPNet model, we are able to comprehend the images more accurately and robustly. According to the results achieved by our proposed approach, it is quite obvious that the suggested approach shows great promise for utilizing Pap smear images to determine the extent of dysplasia present in cervical lesions as well as reduce the time needed for manual observations.

In the future, we will focus on expanding the data set further in order to maintain the constant overall accuracy while keeping images that contain varying degrees of dysplasia. Since, the metaplastic category cells have the lowest accuracy rate, we will concentrate on improving its accuracy in the future. In future, we will also consider combining our work on regularized ViT model variant and feature fusion integration components to build a robust yet efficient model. Additionally, investigating methods for utilizing multimodal data, such as clinical data and patient history may enhance the accuracy and efficacy of cervical cancer diagnosis.

## Data Availability

Publicly available datasets were analyzed in this study. This data can be found here: https://www.kaggle.com/datasets/akshaykrishnan/sipakmed5.
